# Emerging targets for positron emission tomography imaging in proteinopathies

**DOI:** 10.1038/s44303-024-00032-4

**Published:** 2024-08-21

**Authors:** Melissa Chassé, Neil Vasdev

**Affiliations:** 1 Azrieli Centre for Neuro-Radiochemistry, Brain Health Imaging Centre, Centre for Addiction and Mental Health, Campbell Family Mental Health Research Institute, Toronto, ON Canada; 2 Institute of Medical Science, Temerty Faculty of Medicine, University of Toronto, Toronto, ON Canada

**Keywords:** Imaging techniques, Brain imaging

## Abstract

Positron emission tomography (PET) imaging of neurodegenerative disease has historically focused on a small number of established targets. The development of selective PET radiotracers for novel biological targets enables new ways to interrogate the neuropathology of proteinopathies and will advance our understanding of neurodegeneration. This perspective aims to highlight recent PET radiotracers developed for five emerging targets in proteinopathies (i.e., mHTT, BACE1, TDP-43, OGA, and CH24H).

## Introduction

Proteinopathies are a group of neurodegenerative conditions characterized by the formation of misfolded and/or aggregated proteins which progressively spread within the central nervous system (CNS)^1–3^. Despite this shared protein aggregate phenotype, proteinopathies span a large array of clinical presentations, including Alzheimer’s disease (AD), Parkinson’s disease (PD), Huntington’s disease (HD), frontotemporal dementias (FTLDs), amyotrophic lateral sclerosis (ALS), and others. Accurate diagnosis of these conditions can be difficult due to overlapping clinical symptoms in the early stages of the disease, in addition to common co-pathologies and comorbidities^4–10^. As such, specific disease markers, or combinations thereof, are critical for increasing diagnostic accuracy. Early disease markers would also be beneficial for investigating treatment strategies by enabling patient stratification for therapeutic targets and monitoring treatment efficacy in the pre-symptomatic stages of disease when treatments have the potential to be most impactful^10^.

Positron emission tomography (PET) is a highly sensitive functional imaging technique that relies on positron-emitting radionuclides integrated into a molecule of interest (or ‘radiotracer’) to trace its distribution within a living system. When radiotracers have high binding affinities for a biological target associated with disease (or ‘biomarker’), PET imaging can be used to quantify biomarker expression in vivo. PET is a particularly powerful tool for basic science investigations of human neurobiology under homeostatic and pathological conditions, as it allows for biomarker quantification in living subjects. Due to its minimally invasive nature, PET also allows for repeated imaging of the same subjects to examine changes in response to external stimuli or to track biomarker changes longitudinally, which can be used for monitoring disease progression and treatment response.

Given the utility of PET for diagnostic and drug development purposes, it has been consistently used for proteinopathy research for several decades. Out of the 18 PET radiopharmaceuticals approved for clinical use by the United States Food and Drug Administration, five are primarily approved for imaging neurodegenerative diseases including proteinopathy-specific indications (Neuraceq^TM^, Amyvid^TM^, Tauvid^TM^, Vizamyl^TM^, [^18^F]6-Fluoro-L-dopa), and one has seen extensive off-label use for clinical investigations of proteinopathies ([^18^F]FDG)^11^. Development of PET radiotracers for proteinopathy neuroimaging has historically focused on a handful of biological targets, namely protein aggregates of amyloid-beta, hyperphosphorylated tau, and alpha-synuclein^12–16^. In spite of the decades of effort put into imaging these targets, there remain many unanswered questions in the field. For example, there are no disease-modifying treatments for any proteinopathy, nor are there any accepted methods for early diagnosis of sporadic proteinopathies. Development of PET radiotracers to study novel molecular targets in proteinopathies may offer much-needed insights into these conditions.

This perspective focuses on the imaging of five novel biomarkers in neurodegeneration which have seen major developments within the past five years (see Table 1). Importantly, we aspire to highlight promising imaging biomarkers that fall outside of the historically targeted scope of PET imaging of proteinopathies (e.g., amyloid-beta, tau, alpha-synuclein). This perspective is intended to generate enthusiasm for imaging novel targets to study proteinopathies rather than serve as a comprehensive review, which have been published elsewhere^12–22^.

**Table 1:** Proteinopathy imaging targets and associated radiotracers discussed herein

Target	Radiotracers
Mutant huntingtin protein (mHTT)	[^11^C]CHDI-180L
	[^11^C]CHDI-180R
	[^11^C]CHDI-626
	[^18^F]CHDI-650
β-Site amyloid precursor protein cleaving enzyme 1 (BACE1)	[^18^F]PF-06684511
	[^11^C]RO6807936
*O*-linked β-*N*-acetylglucosamine hydrolase (OGA)	[^18^F]MK8553
	[^11^C]LSN3316612
	[^18^F]LSN3316612
	[^11^C]BIO-735
	[^11^C]BIO-578
Cholesterol 24-hydroxylase (CH24H)	[^11^C]**1**
	[^18^F]T-008
	[^18^F]Cholestify
Transactive response DNA binding protein of 43 kDa (TDP-43)	AC Immune Compounds

## Mutant huntingtin protein (mHTT)

Huntington’s Disease is a genetic condition caused by an expanded CAG trinucleotide repeat in the huntingtin gene, resulting in mHTT with an extended poly-glutamine sequence^23^. Expression of pathogenic huntingtin results in intranuclear inclusions of mHTT aggregates and contributes to the loss of excitatory medium spiny neurons in the striatum and cortex. Aggregates of mHTT are sparsely expressed and tend to be much smaller than other protein aggregates, thereby limiting the number of available binding sites. These attributes have contributed to the difficulty in finding CNS PET radiotracers with sufficient binding affinities to provide suitable binding potentials (BP = B_max_/*K*_d_) for imaging (BP > 5) and high selectivity over other CNS targets, including more highly expressed protein aggregates^24^. The focus of this section will be the extensive medicinal chemistry efforts put forth by the CHDI Foundation, which has been leading the way for PET imaging of mHTT.

**Fig. 1:**
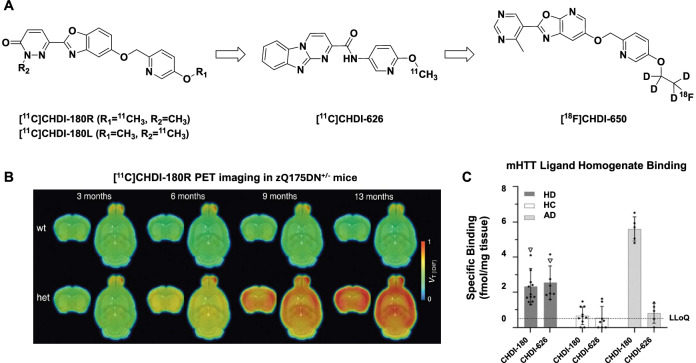
Developments in PET radiotracers for imaging mHTT aggregates. **A** Evolution of CHDI mHTT radiotracers to date. **B** Longitudinal [^11^C]CHDI-180R images in wild-type and zQ175DN heterozygous mHTT expressing mice. Adapted from Bertoglio, D. et al. Development of a ligand for in vivo imaging of mutant huntingtin in Huntington’s disease. *Sci. Transl. Med*. **14**, eabm3682 (2022). Reprinted with permission from AAAS. **C** Quantification of [^3^H]CHDI-180 and [^3^H]CHDI-626 binding in HD, healthy control (HC) and AD human brain homogenates. Adapted from Herrmann, F. et al. Pharmacological characterization of mutant huntingtin aggregate-directed PET imaging tracer candidates. *Sci. Rep.* **11**, 17977 (2021).

The first reported mHTT radiotracers by the CHDI were isotopologues of CHDI-180, distinguished by “left-side” *O*-methylation ([^11^C]CHDI-180L) or “right-side” *N*-methylation ([^11^C]CHDI-180R) (Fig. 1A)^25^. CHDI-180 is a high-affinity (*K*_d_ = 1–6 nM) ligand specific for mHTT aggregates over normal HTT, monomeric soluble mHTT, or nuclear inclusion bodies^24–26^. [^11^C]CHDI-180R was deemed to be more promising than [^11^C]CHDI-180L due to lower uptake variability and faster washout kinetics when imaging in healthy non-human primates (NHPs)^25^. [^11^C]CHDI-180R was used to detect temporal dynamics of mHTT pathology and responses to treatment in mHTT mouse models (Fig. 1B)^26^. Subsequent autoradiography (ARG) studies with [^3^H]CHDI-180R exhibited significant off-target binding to other protein aggregates in postmortem AD brains (5.6 fmol/mg in AD cortex vs 2.0 fmol/mg in HD cortex) (Fig. 1C)^24,27^. To address this limitation, [^11^C]CHDI-626 (*K*_d_ = 4–6 nM) (Fig. 1A), a structurally related tritiated analog demonstrated high specific binding to mHTT with reduced binding to postmortem AD brains (0.8 fmol/mg), was developed^23,27^. In radioligand binding assays using AD brain homogenates, CHDI-626 showed limited displacement of [^3^H]T807 (a.k.a. Tauvid™, flortaucipir, AV-1451) and [^3^H]flutemetamol (Vizamyl^TM^; IC_50_ > 1 µM), indicating low off-target binding to tau and amyloid-beta aggregates, respectively. PET imaging with [^11^C]CHDI-626 in healthy NHPs showed similar in vivo properties to [^11^C]CHDI-180R with a maximum whole-brain uptake of 4.0 standardized uptake value (SUV), followed by rapid washout and no confounding metabolites.

While both [^11^C]CHDI-180R and [^11^C]CHDI-626 have been advanced for first-in-human use and clinical trials (NCT03810898)^28^, medicinal chemistry efforts continued to enable fluorine-18 labeling and increase the plasma half-lives of these radiotracers to boost target engagement. To that end, the authors developed [^18^F]CHDI-650 (Fig. 1A)^29^. This new radiotracer displayed similar affinity for mHTT aggregates to CHDI-180R and CHDI-626 in saturation binding assays (IC_50_ ~1 nM), while halving the level of off-target binding observed in AD brain homogenates compared to CHDI-180R. Notably, CHDI-650 is marginally less selective than CHDI-626 in AD brain homogenate radioligand binding assays (26% CHDI-650 binding versus 16% CHDI-626 binding). [^18^F]CHDI-650 also exhibited lower peak cortical uptake (2.5 SUV) in NHPs than [^11^C]CHDI-626 (4.0 SUV), but higher than [^11^C]CHDI-180R (2.8 SUV). Metabolite-corrected arterial input functions were used for tracer kinetic modeling; the data fit well with a two-tissue compartment model (2TCM) and confirmed homogeneous distribution and reversible kinetics, in line with the previously reported tracers. The authors state that this radiotracer will be translated for human PET imaging studies despite the more promising characteristics of [^11^C]CHDI-626 (e.g., off-target AD binding, cortical uptake), but this is likely due to the longer-lived fluorine-18 radionuclide which would enable multi-center clinical trials.

## β-Site amyloid precursor protein cleaving enzyme 1 (BACE1)

BACE1 cleaves amyloid precursor protein, making it responsible for the generation of monomeric amyloid-beta^30^. Consequently, abnormal BACE1 activity has been linked to the formation of amyloid-beta plaques. Given the repeated failures of Aβ-targeted clinical trials to demonstrate significantly improved cognition in AD patients, much interest has been placed on BACE1 to better understand the upstream biology of amyloid plaque formation^31^.

**Fig. 2:**
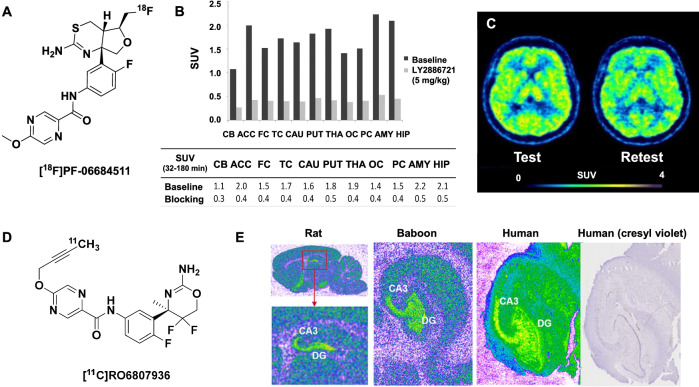
Reported PET radiotracers developed for imaging BACE1. **A** Structure of [^18^F]PF-06684511. **B** Averaged 32–180 min SUVs for [^18^F]PF-06684511 at baseline and under heterologous blocking with 5 mg/kg LY2886721 (PO, 2 h prior to PET scanning). CB cerebellum, ACC anterior cingulate cortex, FC frontal cortex, TC temporal cortex, CAU caudate, PUT putamen, THA thalamus, OC occipital cortex, PC parietal cortex, AMY amygdala, HIP hippocampus. Adapted with permission from Zhang, L. et al. Identification of a novel positron emission tomography (PET) ligand for imaging β-site amyloid precursor protein cleaving enzyme 1 (BACE1) in brain. *J. Med. Chem*. **61**(8), 3296–3308 (2018). Copyright 2018 American Chemical Society. **C** Representative summed [^18^F]PF-06684511 PET images (0–123 min) for test-retest scans in healthy volunteers. Adapted with permission from Arakawa, R. et al. PET imaging of beta-secretase 1 in the human brain: radiation dosimetry, quantification, and test-retest examination of [^18^F]PF-06684511. *Eur. J. Nucl. Med. Mol. Imaging* **47**, 2429–2439 (2020). **D** Structure of [^11^C]RO6807936. **E** In vitro autoradiographical distribution of [^3^H]RO6807936 binding (1 nM) in rat brain, as well as baboon and human hippocampal sections. Neuronal staining with cresyl violet in human hippocampal tissue is also shown. DG dentate gyrus. Adapted with permission from Honer, M. et al. RO6807936 as a novel positron emission tomography (PET) radiotracer for in vitro and in vivo visualization and quantification of beta-site amyloid precursor protein cleaving enzyme (BACE1) in the rodent and baboon brain. *J. Label. Compd. Radiopharm*. **66**, 222–236 (2023).

Researchers at Pfizer were the first to report PET imaging of BACE1 with [^18^F]PF-06684511 (Fig. 2A)^32^. In whole-cell assays, PF-06684511 displayed IC_50_<1 nM and was 15 times more selective for BACE1 over BACE2. Time-activity curves from NHP imaging with [^18^F]PF-06684511 were not initially reported, but a subsequent quantitation study with this tracer revealed relatively slow kinetics, with a peak whole-brain uptake of 2.2 SUV at 20 min, which was halved to 1.0 SUV by 120 min^33^. Heterologous blocking with 5 mg/kg LY2886721 (intravenously, 2 h pre-scan) reduced the 32–180 min averaged [^18^F]PF-06684511 uptake by 48–80% in NHPs, depending on the brain region (Fig. 2B). The radiotracer was advanced to healthy human volunteers^34^. A 2TCM fits both the NHP and human PET data well, although the authors suggest that Logan's graphical analysis may be a suitable alternative^33,34^. No reference regions can be used when imaging this target because BACE1 is ubiquitously expressed throughout the brain. Unfortunately, the measured test-retest variability in healthy human volunteers was determined to be ~16% (Fig. 2C), so a >20% change in V_T_ is necessary to measure significant BACE1 inhibition^34^. The high test-retest variability, paired with the slow washout kinetics, are limiting factors in the use of [^18^F]PF-06684511 for clinical research. Nonetheless, [^18^F]PF-06684511 remains the most advanced PET radiotracer for imaging BACE1 to date.

The most recent attempt to develop a BACE1 imaging agent was published by Roche with [^11^C]RO6807936 (Fig. 2D)^35^. In vitro assays reported high affinity (*K*_d_ = 5.6 nM), moderate selectivity over BACE2 (>16-times) and other aspartyl proteases, weak P-glycoprotein efflux (efflux ratio 1.5), and suitable effective permeability (117 nm/s). In spite of these promising in vitro results, [^11^C]RO6807936 demonstrated poor brain uptake in NHPs (<1.0 SUV at 10 min post-injection), thereby limiting its use for in vivo imaging. This radiotracer also exhibited rapid and extensive radio metabolism, although that is not surprising given the high clearance reported in microsomes and hepatocytes. To our knowledge, this work represents the first BACE1 distribution reported using in vitro ARG (Fig. 2E).

## *O*-linked β-*N*-acetylglucosamine hydrolase (OGA)

*O*-linked β-*N*-acetylglucosamine (*O*-GlcNAc) hydrolase (OGA) modulates intracellular protein function through the removal of O-GlcNAc from post-translationally glycosylated serine and threonine residues. Disruption of homeostatic OGA function has been suggested to play a role in various neurodegenerative conditions due to the presence of O-GlcNAc on relevant proteinopathy biomarkers, including APP, tau, α-synuclein, superoxide dismutase, and neurofilament proteins, which are involved in ALS and other neuropathologies^36,37^.

**Fig. 3:**
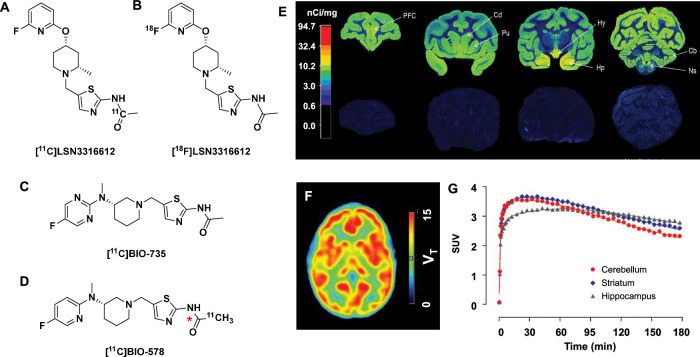
PET radiotracers for imaging OGA expression. Structures of **A** [^11^C]LSN3316612, **B** [^11^C]LSN3316612, **C** [^11^C]BIO-735, and **D** [^11^C]BIO-578. **E** In vitro ARG in NHP brain tissue with [^3^H]LSN3316612 (5 nM) at baseline (top column) or after 20 μM thiamet G blocking (bottom columns) with indicated brain regions. Cb cerebellum, Cd caudate nucleus, Hp hippocampus, Hy hypothalamus, Ns substantia nigra, PFC prefrontal cortex, Pu putamen. **F** Representative derived parametric brain images (summed 0 to 180 min) in a healthy volunteer with [^18^F]LSN3316612. **G** Representative regional time-activity curves of [^18^F]LSN3316612 in a healthy volunteer. Adapted from Lu, S. et al. PET ligands [^18^F]LSN3316612 and [^11^C]LSN3316612 quantify *O*-linked-β-*N*-acetyl-glucosamine hydrolase in the brain. *Sci. Transl. Med*. **12**, eaau2939 (2020). Reprinted with permission from AAAS.

The first PET radioligand for OGA reported by Merck, [^18^F]MK8553, was disclosed in a conference abstract^38^. The first published articles for brain imaging of OGA discussed the development of [^3^H]/[^11^C]/[^18^F]LSN3316612 (OGA IC_50_ = 1.91 nM) (Fig. 3A, B)^39^. Saturation binding experiments and autoradiography with [^3^H]LSN3316612 performed in rats, cynomolgus monkeys, and human brain homogenates resulted in calculated BPs ranging from 2.2 in NHP cerebellum, up to 22.9 in human striatum (Fig. 3E). Both [^11^C]LSN3316612 and [^18^F]LSN3316612 reversibly bound OGA in NHP brain when imaged in vivo, with regional uptake that correlated well with OGA enzyme distribution and that could be heterologously blocked by thiamet G (10 mg/kg, i.v., 45 min pre-injection). [^11^C]LSN3316612 was not pursued past NHP imaging because of its low radiochemical yields (<5%), but [^18^F]LSN3316612 was used to image 8 healthy human participants with high peak brain uptake (~4 SUV) (Fig. 3F, G). Both NHPs and human scans reached a stable V_T_ as determined by a 2TCM with arterial input functions, indicating that the observed polar radiometabolites (~60% at 60 min) do not interfere with quantification. While initial results with [^18^F]LSN3316612 were promising, the test-retest reliability was modest and inexplicably demonstrated increased V_T_ on afternoon retest scans in almost all participants, potentially complicating its clinical utility^40,41^. Moreover, relatively slow clearance kinetics make robust quantification difficult due to the lack of a reference region.

Subsequently, a structurally similar pair of carbon-11 methylated radiotracers, [^11^C]BIO-735 and [^11^C]BIO-578 were described (Fig. 3C, D)^42^. Radioligand binding assays performed with [^3^H]BIO-735 in rat brain regional homogenates and human frontal cortex homogenate had a K_d_ of 0.6 nM, with regional differences in B_max_ in accordance with known differences in OGA concentration. Interestingly, the human frontal cortex had a B_max_ of 39 nM, but the analyzed human T-cell homogenate was found to have a B_max_ of 42 nM. Binding to circulating T cells may present a challenge for quantification due to: (1) peripheral blocking causing changes in the tracer-free fraction and (2) typical input function analysis would not account for tracer binding to circulating T cells. In vivo imaging in rodents and NHPs demonstrated rapid brain uptake and good specific binding of [^11^C]BIO-735, but with little discernible washout during the scan duration. In light of the slow off-rate for [^11^C]BIO-735 to OGA, the authors developed [^11^C]BIO-578 to reduce OGA affinity (rodent *K*_d_ = 2.3 nM) for accelerated clearance without affecting selectivity or specificity. In vivo NHP imaging with [^11^C]BIO-578 showed similar uptake and regional distribution (putamen SUV_max_ ~5), but with improved washout kinetics. Heterologous blocking with 10 mg/kg thiamet G resulted in significant clearance from all brain regions, indicating that the observed brain uptake was selective. A carbon-11 carbonyl isotopologue synthesized via [^11^C]CO demonstrated identical imaging characteristics and similar metabolic stability (i.e., 15% versus 10% intact [^11^C]BIO-578 in the plasma at 60 min)^43^.

## Cholesterol 24-hydroxylase (CH24H or CYP46A1)

CH24H, also known as CYP46A1, is a monooxygenase in the cytochrome P450 family responsible for neuronal cholesterol metabolism to 24-hydroxycholesterol. Abnormalities in cholesterol homeostasis have been associated with neurological conditions, including a range of neurodegenerative diseases, including AD, PD, and HD^44^. Given that CH24H is primarily responsible for removing excess cholesterol from the brain, its dysfunction may contribute to the accumulation of cholesterol and its byproducts in neurons. Therefore, PET radiotracers for measuring CH24H expression as a proxy for brain cholesterol metabolism in vivo are of interest for clinical research.

**Fig. 4:**
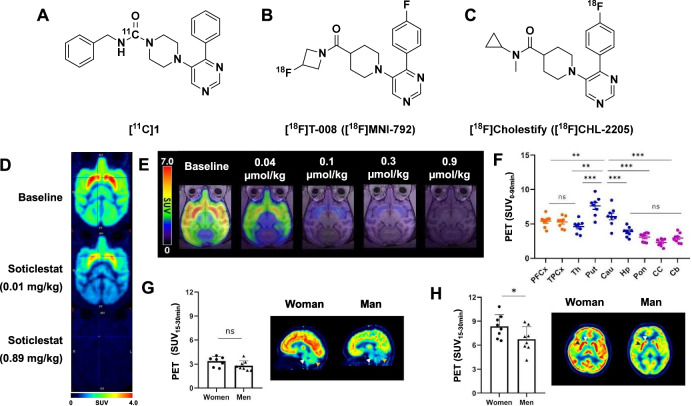
Radiotracers reported for imaging CH24H expression and function. Structures of CH24H radiotracers **A** [^11^C]1, **B** [^18^F]T-008 (aka [^18^F]MNI-792), and **C** [^18^F]Cholestify (aka [^18^F]CHL-2205). **D** Summed [^18^F]T-008 PET images (0–180 min) in rhesus macaque at baseline and post dosing with soticlestat at 0.01 and 0.89 mg/kg. Adapted from Koike, T. et al. Preclinical characterization of [^18^F]T-008, a novel PET imaging radioligand for cholesterol 24-hydroxylase. *Eur. J. Nucl. Med. Mol. Imaging* **49**, 1148–1156 (2022). **E** Target occupancy study with [^18^F]Cholestify in rhesus macaque using different doses of soticlestat. **F** Quantitative regional SUVs (average 0–90 min) from healthy human scans (PFCx prefrontal cortex, TPCx temporal cortex, Th thalamus, Put putamen, Cau caudate, Hp hippocampus, Pon pons, CC corpus callosum, Cb cerebellum). **G** Female and male [^18^F]Cholestify uptake (average SUVs 15–30 min) in low CH24H regions, cerebellum (brown arrow) and brainstem (white arrow). **H** Female versus male [^18^F]Cholestify uptake (average SUVs 15–30 min) in high CH24H regions, caudate (white arrow) and putamen (black arrow). *P < 0.05, **P < 0.01, and ***P < 0.001. [^18^F]Cholestify images adapted from Ahmed Haider et al. Assessment of cholesterol homeostasis in the living human brain. *Sci. Transl. Med*. **14**, eadc9967 (2022). Reprinted with permission from AAAS.

The first radiotracer developed for this target ([^11^C]**1**) had low potency (*K*_i_ = 7.3 µM), poor rodent peak brain uptake (0.4 SUV), and specificity in vivo (Fig. 4A)^45^. [^18^F]T-008, also known as [^18^F]MNI-792, was later reported as having high affinity (*K*_d_ = 14 nM) and specific binding to CH24H (Fig. 4B)^46,47^. ARG studies with [^3^H]T-008 did not show significant radioligand binding in CH24H knock-out mice. In wild-type mouse brain sections, [^3^H]T-008 demonstrated 80% specific binding when heterologously blocked with CH24H inhibitor soticlestat. [^18^F]T-008 displayed heterogeneous uptake in NHP PET scans which aligned with the expression levels seen in rodent tissue staining and ARG. The caudate and putamen had the highest uptake (>4 SUV at 30 min), and the cerebellum had the lowest (2.5 SUV at 5 min). Blocking studies with soticlestat reduced maximum uptake and increased washout kinetics in all brain regions in a dose-dependent manner, indicating that this tracer can reasonably be used for target occupancy studies (Fig. 4D). Dose-dependent blocking was even observed in CH24H-poor regions, such as the cerebellum, indicating that there are no appropriate reference regions within the brain for CH24H imaging. As such, the V_T_ was estimated via Logan graphical analysis (LGA) using an arterial-plasma input function with an average test-retest variability was <20%.

A structurally similar radiotracer, [^18^F]Cholestify (a.k.a [^18^F]CHL-2205), was reported by Haider and coworkers (Fig. 4C)^48,49^. Binding assays in rodent brain homogenate provided a *K*_d_ of 0.36 nM, with B_max_ values ranging from 117 fmol/g in the striatum, down to 22 fmol/g in the cerebellum. ARG studies show that [^18^F]Cholestify binds CH24H-rich areas in rodent and NHP brain sections (e.g., striatum and cortex), with demonstrably less uptake in low abundance areas (e.g., cerebellum). The selectivity of [^18^F]Cholestify for CH24H was demonstrated through biochemical affinity screening assays, ARG blocking with soticlestat (87% decrease in binding), and PET imaging in CH24H knock-out mice, which showed almost no radiotracer uptake. PET imaging in healthy rodents and NHPs displayed rapid, heterogeneous brain uptake in accordance with CH24H expression, slow radiotracer washout, and no significant brain-penetrant radiometabolites. Mass spectrometry in rodent tissue confirmed that ex vivo levels of 24-hydroxycholesterol strongly correlated with radiotracer uptake in vivo (*R*^2^ = 0.893), but not with other neuronal metabolites, confirming that [^18^F]Cholestify can be used to quantify levels of neuronal cholesterol metabolism. Much like previously described for [^18^F]T-008, soticlestat blocking studies in NHPs resulted in dose-dependent signal attenuation across all brain regions (Fig. 4E). In human volunteers, [^18^F]Cholestify uptake reflected CH24H-rich brain regions and scan times could accurately quantify CH24H expression in only 15 min (Fig. 4F). Interestingly, there were no significant sex-differences in radiotracer uptake in target-poor regions (Fig. 4G), but women exhibited higher uptake in target-rich regions compared to men (Fig. 4H). Given that women are more likely to be diagnosed with AD than men of the same age^50^, these findings may dovetail well with the preclinical [^18^F]Cholestify PET imaging that these authors did in 3xTg-AD mice, which showed that the AD mice had higher specific hippocampal uptake compared to control animals^49^.

Radiotracers for quantifying neuronal cholesterol metabolism via CH24H expression levels should prove to be an asset for the neuroscience community in the study of various neuropathologies. Of particular interest will be the further investigation of these preliminary sex-based differences in CH24H levels of healthy human volunteers, and how the difference in homeostatic CH24H levels may play a part in sex-based incidence rates of neurodegeneration and other cholesterol-related CNS disorders.

## Transactive response DNA binding protein of 43 kDa (TDP-43)

Intracellular aggregates of TDP-43 in neurons and oligodendrocytes have been identified in familial ALS and FTLDs^51,52^. These amyloid-like filamentous aggregates have historically been a highly sought target of CNS PET radiotracer design. Despite several attempts to develop a promising PET radiotracer for TDP-43, little progress has been made for human translation^53,54^. An abstract by AC Immune from the conference proceedings of Alzheimer’s Association International Conference 2023 (AAIC 2023) by Seredinina et al announced the discovery of a first-in-class series of tritiated radiotracers with nanomolar affinity and high selectivity for TDP-43 over other protein aggregates, as identified through radioligand binding assays in FTLD-TDP, AD, and PD postmortem human brain tissue sections^55^. Lead compounds were radiolabeled with fluorine-18 and reported to show fast uptake and subsequent washout in healthy NHPs. Once formally reported, these radiotracers have the potential to offer tremendous advancements for TDP-43-related diagnostics and treatments.

## Conclusions and future outlook

The development of PET probes for novel biological targets in neurodegeneration is an exciting area of research with untold possibilities. We are pleased to see that in recent years, a rapidly growing number of novel targets have become of interest for imaging proteinopathies. While undoubtedly a challenging and resource intensive endeavor, with increasing regulatory burdens and costs, developing imaging agents for novel biomarkers has the potential to not only improve our academic understanding of neurodegenerative disease, but also have a real lasting impact on clinical diagnosis and treatment.
